# The influence of physical education courses integrated with civic education on prosocial behavior among college students: the chain mediating effect of cultural confidence and self-esteem

**DOI:** 10.3389/fpsyg.2023.1217290

**Published:** 2023-11-02

**Authors:** Changchang Huang, Geng Li, Yuantong Zhang, Nalatporn Aphichaithawon, Zhile Deng, Zhihua Zhang, Yihan Zhang, Jianjun Ding

**Affiliations:** ^1^Hunan Sany Polytechnic College, Changsha, China; ^2^College of Physical Education, Hunan Normal University, Changsha, China; ^3^Rausser College of Natural Resources, University of California, Berkeley, Berkeley, CA, United States; ^4^Shaoyang No. 10 Middle School, Shaoyang, China; ^5^School of Educational Science, Hunan Normal University, Changsha, China; ^6^College of Marxism, Hunan Normal University, Changsha, China

**Keywords:** physical education, prosocial behavior, college student, cultural confidence, self-esteem

## Abstract

**Objective:**

This study sought to uncover the relationship between physical education courses integrated with civic education (PECICE) and prosocial behavior among university students. Additionally, we aimed to decipher the mediating roles of cultural confidence and self-esteem within this relationship.

**Methods:**

Employing a questionnaire-based approach, we assessed the relationship among PECICE, cultural confidence, self-esteem, and prosocial behavior in university students. The instrument comprised four distinct scales: the Perceived Effectiveness Scale for PECICE, the Cultural Confidence Scale, the Self-Esteem Scale, and the Prosocial Behavior Scale. Our sample encompassed 293 Chinese college students, consisting of 137 men and 156 women, with an average age of 21.39 years (SD = 2.1).

**Results:**

PECICE demonstrated significant positive associations with cultural confidence (*r* = 0.29, *p* < 0.001), self-esteem (*r* = 0.35, *p* < 0.001), and prosocial behavior (*r* = 0.40, *p* < 0.001). The influence of PECICE on prosocial behavior among university students was mediated through three channels: solely via cultural confidence (mediating effect value: 0.14), solely via self-esteem (mediating effect value: 0.22), and through the combined influence of both cultural confidence and self-esteem (mediating effect value: 0.2).

**Conclusion:**

The intertwined mediating roles of cultural confidence and self-esteem highlight their pivotal significance in enhancing the efficacy of PECICE. These insights offer a valuable reference for both educators and policymakers striving to augment prosocial behavior in university students.

## 1. Introduction

Prosocial behavior, encompassing actions such as helping, cooperating, sharing, and comforting, serves as a cornerstone for harmonious social development (Campbell et al., 1999; Eisenberg et al., 2006). Such behaviors not only promote positive emotions, provide social support, and mitigate stress, but they also bestow benefits upon both the helper and the recipient (Raposa et al., 2016; American Psychological Association [APA], 2020). Furthermore, nurturing prosocial behaviors is essential in cultivating a culture saturated with empathy and social responsibility, which in turn enhances the communal well-being and success (Carlo et al., 2011). For university students, positioned between adolescence and early adulthood, this phase marks a critical juncture in the molding of their social identities and ethical compasses (Wang et al., 2021). Research has demonstrated a connection between prosocial behavior and aspects such as academic achievement, social interactions, and overall physical and mental health among university students (Layous et al., 2012; García-Hermoso et al., 2021). Recognizing its profound implications, it becomes imperative to comprehend the nuances of prosocial behavior’s evolution within this demographic. Consequently, this research endeavors to delve into the developmental trajectories of prosocial behavior among university students, aiming to provide tailored strategies to nurture such behaviors.

### 1.1. Literature review in prosocial behavior

Research on prosocial behavior has extensively focused on both internal and external factors (Malti et al., 2016; Hughes et al., 2018). Internally, individual attributes, such as healthy self-esteem, narcissism, empathy, and a sense of security, have been found to bolster prosocial behavior (Moscardino et al., 2020; Pang et al., 2022). Externally, family dynamics, particularly parenting styles, greatly influence this behavior. Notably, while authoritarian, indulgent, and neglectful parenting styles tend to diminish prosocial behavior, positive parenting approaches enhance it (Lin and Shan, 2022). However, the socio-ecological theory posits that the interplay between individuals and their surrounding socio-ecological systems shapes their psychological and behavioral trajectories (Bronfenbrenner, 1974). Among these systems, schools, especially during university years, are pivotal. They are venues where individuals assimilate values, attitudes, cognitive styles, and behaviors through observation and learning (Bonell et al., 2013). Despite its significance, few studies have delved into the influence of the school environment on the prosocial behavior of university students.

For university students, school curricula serve as foundational platforms to forge interpersonal relationships and social networks. Such curricula are often credited for the positive influence schools exert on prosocial behavior (Mendonça et al., 2014; Crone and Achterberg, 2017; Li and Shao, 2022; Yao and Enright, 2022). Recently, Chinese scholars have taken a keen interest in melding physical and civic education (Wang, 2019; Zhao et al., 2021; Hu D. P., 2022). Physical education, with its inherent structure that promotes social interactions, teamwork, and conflict resolution, is posited to amplify prosocial behavior among students (Bailey et al., 2009). On the other hand, civic education equips students with the knowledge and experiences necessary for active participation in democratic processes (Rietbergen-McCracken, 2012). Such an education accentuates the understanding of societal issues and spurs active community engagement, further fostering prosocial behavior (Vasiljevic, 2009). Nonetheless, the confluence of physical and civic education in nurturing prosocial behavior remains an under-researched area. Thus, delving into the combined influence of these two disciplines on prosocial behavior is crucial.

### 1.2. Physical education courses integrated with civic education and prosocial behavior

The integration of civic education elements into physical education courses, termed as physical education courses integrated with civic education (PECICE), aims to inculcate in students’ values such as patriotism, collectivism, legal awareness, and moral integrity (Fu and Ding, 2008; Zhao et al., 2021). Recognized as a vital strategic step toward enriching physical education reforms and enhancing civic education quality, PECICE has been progressively institutionalized in Chinese higher education (Hu X., 2022). There are predominantly two methodologies underpinning PECICE (Wang, 2019). The first, termed the “integrative” approach, weaves content that resonates with physical education, like notable figures, historic moments, and events, into the curriculum. An example is Tianjin Sports College, which infuses attributes exemplified by the Chinese women’s volleyball team, such as patriotism and resilience, into its teachings. The second, known as the “explorative” approach, draws out civic values inherent in professional course content or specialized physical education techniques. During classes that touch upon topics like sports etiquette or the dynamics of student interactions in sports settings, educators might underscore civic virtues like resilience, collaboration, responsibility, and moral judgment. Given that PECICE’s pedagogical goals resonate deeply with the values advocated by prosocial behavior, this study proposes hypothesis 1 that PECICE holds the potential to be a significant predictor of university students’ prosocial behavior.

### 1.3. The mediating effect of cultural confidence

The interplay of external environment and individual intrinsic factors jointly steers the course of psychological and behavioral development (Magnusson and Stattin, 1996). Thus, for an external stimulus like PECICE to effectively stimulate prosocial behavior, it needs to synchronize with intrinsic qualities. Among such qualities, cultural confidence—a profound sense of cultural identity and belonging—stands out (Pan et al., 2021). Cultural confidence not only correlates with prosocial behavior but may also be a pivotal internal factor driving it (Irwin, 2009; Zhang et al., 2020). People with a strong sense of cultural confidence typically display altruistic tendencies and cooperate with those sharing similar cultural backgrounds (Zhang et al., 2020). Building on the Group Selection Theory (Wilson, 1975), in-group members often elicit greater cooperation and are more likely to receive assistance when in need compared to out-group members (De Dreu, 2010; Tseng et al., 2011). Such favoritism is attributed to perceived similarities with in-group members, which bolsters prosocial actions. Stürmer et al. (2006) further delineated group affiliation influences altruistic decisions across varied cultural contexts. In this light, cultural confidence appears to reinforce the group selection mechanism, fortifying prosocial behavior.

Grounded in the Social Learning Theory (Bandura and Walters, 1977), individuals’ beliefs and behaviors are shaped by observing and mirroring their surroundings. Education, a pivotal societal construct, plays an instrumental role in sculpting students’ cognition, values, and ethos (Sun and Ren, 2022). This underscores the potential of curricula in nurturing cultural confidence. Empirical findings corroborate that civic education fosters cultural confidence by encouraging an exploration of diverse cultural spectra (Xu and Yu, 2014). However, the link between PECICE and cultural confidence remains under-explored. In light of the aforementioned evidence, it is plausible that PECICE could influence cultural confidence. Consequently, this study proposes hypothesis 2 that cultural confidence may mediate the relationship between PECICE and prosocial behavior in university students.

### 1.4. The mediating effect of self-esteem

Beyond the realm of cultural confidence, self-esteem emerges as a salient intrinsic factor that profoundly influences individual behaviors. Defined as an individual’s introspective evaluation of their intrinsic value (Orth and Robins, 2014), self-esteem plays a cardinal role in steering one’s psychological trajectory, encompassing areas such as prosocial tendencies and ethical disposition (Qian and Xiao, 1998). A plethora of empirical studies underscore the significance of high self-esteem as a harbinger of prosocial behavior, suggesting that individuals with robust self-worth are predisposed to altruistic actions (Damon et al., 2006; Hu and Ding, 2011). In essence, elevated self-esteem engenders a benevolent self-perception. When individuals resonate with a fortified self-image and exude confidence in their inherent value, they tend to display prosocial behaviors that align harmoniously with their internal schema (Jiemin et al., 2023). Moreover, contemporary research propounds that individuals with a pronounced self-regard exhibit heightened empathy, rendering them more attuned to the emotional landscapes of others (Gan et al., 2014). This amplified empathetic resonance predisposes them to respond more compassionately to the vicissitudes of others, fostering a greater propensity for prosocial behavior (Zhang and Zhang, 2015). Consequently, self-esteem emerges not merely as an individualistic trait but as a linchpin bolstering an individual’s inclinations toward prosocial actions.

Drawing upon the Self-Determination Theory (Deci and Ryan, 2012), intrinsic psychological needs such as autonomy, competence, and relatedness emerge as pivotal determinants of an individual’s cognitive framework and subsequent actions. It is intriguing to consider how PECICE interfaces with self-esteem through the lens of this theory. At its core, civic education that fosters critical reasoning, judicious decision-making, and vocal articulation of perspectives inherently nourishes autonomy (Brighouse, 1998). When dovetailed with physical education, students’ agency to elect their preferred physical activities augments feelings of competence and autonomy, serving as an impetus for heightened self-esteem (Teixeira et al., 2012). Amplifying this, PECICE frequently integrates collaborative components such as group discourses, joint projects, and collective engagements, scaffolding a platform for students to weave meaningful social fabric and immerse in relatedness-driven interactions (Zhao et al., 2021). Thus, PECICE, by crafting a more resonant learning environment, amplifies the pragmatic application of civic principles by students. While the tangible effect of PECICE on boosting self-esteem remains an undercharted territory, the underpinnings of the Self-Determination Theory provide compelling breadcrumbs hinting at PECICE’s instrumental role in sculpting prosocial dispositions among tertiary students. Anchored in these insights, this study proposes hypothesis 3 that self-esteem could emerge as a pivotal mediator bridging the dynamics between PECICE and the prosocial orientations of university students.

### 1.5. The chain mediating effect of cultural confidence and self-esteem

From the preceding discourse, the instrumental roles of cultural confidence and self-esteem as potential mediators between PECICE and the prosocial inclinations of university students come to the fore. A salient revelation from contemporary research underscores the potency of cultural confidence as a linchpin in the evolution of self-esteem (Hu D. P., 2022). As a corollary, PECICE might sculpt the prosocial contours of university students through a chain mediation rooted in cultural confidence and subsequently channeled through self-esteem. Turning to the Social Identity Theory (Hogg, 2016), it posits that individuals, when resonating with a particular social collective, assimilate the group’s attributes, ethos, and identity into their self-schema. Research echoes that individuals anchored in a robust cultural mooring are predisposed to elevated self-esteem (Sellers and Shelton, 2003). In juxtaposition, any semblance of a cultural identity being in the crosshairs of prejudice or discrimination may cast shadows on their self-esteem, thereby influencing their holistic well-being (Crocker and Major, 1989). Given the relationship between cultural confidence and self-esteem, this study proposes hypothesis 4 that cultural confidence and self-esteem have a chain mediating role in the process by which PECICE influences the prosocial behavior of university students.

To encapsulate, while the beneficial impact of PECICE on the prosocial orientations of university students finds resonance in theoretical discourse, empirical probes into this realm remain relatively nascent. This endeavor seeks to embark on a rigorous exploration of the nexus between PECICE and the prosocial behavior of university students, whilst delving into the chain mediating role of cultural confidence and self-esteem. By charting this terrain, the research aspires not merely to discern the intricate interrelationships but also to offer a compass, both theoretically and empirically, for the future cultivation and augmentation of prosocial predispositions in the university echelons.

## 2. Materials and methods

### 2.1. Procedures and participants

To ensure the questionnaire’s quality and minimize any potential influence on participants, we invited three experts in both psychology and education to evaluate the content of the questionnaire before distribution. Participants were recruited via Wenjuanxing’s online survey platform to complete the questionnaire. Data were collected using convenience sampling from several universities located in the Hunan, Guangdong, Hubei, and Anhui regions of China in April 2023. The study employed standardized guidelines to instruct participants to complete the questionnaire according to their actual situation, and all participants provided voluntary and anonymous informed consent. The questionnaire in this study includes four scales, including the Perceived Effectiveness Scale for PECICE, the Cultural Confidence Scale, the Self-Esteem Scale, and the Prosocial Behavior Scale.

A total of 375 Chinese university students were recruited, whereas 82 of them were excluded because of the following reasons: (1) 53 participants were excluded because of selecting the same response option for every question; (2) 21 participants were excluded because their answering times were <200 s; and (3) 8 participants were excluded because of failing to correctly answer one or more attention check questions. Therefore, 293 participants (137 men and 156 women, *M* = 21.39 years, SD = 2.1) were ultimately retained with an effective rate of 78.13% (Table 1). Among them, 49 were freshmen, 43 were sophomores, 39 were juniors, and 51 were seniors, accounting for 16.7, 14.7, 13.3, and 17.4% of the total sample, respectively. Additionally, there were 61 first-year graduate students, comprising 20.8% of the total, 28 second-year graduate students, representing 9.6% of the total, and 22 third-year graduate students, making up 7.5% of the total.

**TABLE 1:** The demographics of the participants.

Variables	Categories	Number of participants	Percentage (%)
Gender	Male	137	46.8
	Female	156	53.2
Grade	Freshman	49	16.7
	Sophomore	43	14.7
	Junior	39	13.3
	Senior	51	17.4
	First-year graduate student	61	20.8
	Second-year graduate student	28	9.6
	Third-year graduate student	22	7.5
Age	18	34	11.6
	19	38	13
	20	35	11.9
	21	41	14
	22	36	12.3
	23	57	19.5
	24	32	10.9
	25	20	6.8

### 2.2. Measurements

#### 2.2.1. PECICE

The measurement for PECICE was conducted using the “Perceived Effectiveness Scale for PECICE.” This scale is an adaptation of the “Effective Classroom Environment Evaluation Scale for Foreign Language Courses Integrated with Civic Education” by Zhou et al. (2022), which has been proven to have good reliability and validity in a Chinese context in peer-reviewed papers (Qin et al., 2023). Based on the characteristics of the physical education course and expert consultation and recommendations, adjustments were made to the items. For instance, “The civic element is well-represented in foreign language lessons” was modified to “The civic element is well-represented in physical education lessons.” The scale consists of 9 items and is a self-report measure regarding the richness of learning, psychological support, and environmental support of PECICE. Sample items include: “Civic education content is better immersive and embodied,” “Teaching content with a civic education theme,” etc. Each item uses a 5-point Likert scale, with 1 being “Strongly Disagree” and 5 being “Strongly Agree.” The total score ranges from 9 to 45. A higher score indicates that the participants have a stronger perception of the civic education elements during the physical education course. In other words, the participants feel a stronger curriculum atmosphere promoting civic education elements in the physical education course. In this study, the Cronbach’s α coefficient for this scale was 0.85.

#### 2.2.2. Cultural confidence

The measurement for cultural confidence was conducted using the “Cultural Confidence Scale.” The Cultural Confidence Scale was developed by Zhou and Bi (2020) and has been demonstrated to possess good reliability and validity within the Chinese context in peer-reviewed papers (Wang and Jia, 2021). The scale consists of 10 items that measure an individual’s cultural confidence, cultural confidence motivation, and cultural confidence emotion. Sample items include: “I’m proud of Chinese culture,” and “Chinese culture has unique values and is not inferior to foreign cultures.” Each item utilizes a 7-point Likert scale, with 1 being “Strongly Disagree” and 7 being “Strongly Agree.” The total score ranges from 7 to 70, and a higher score indicates a higher degree of cultural confidence. In this study, the Cronbach’s α coefficient for this scale was 0.89.

#### 2.2.3. Self-esteem

The measurement for self-esteem was conducted using the “Self-Esteem Scale.” The Self-Esteem Scale was developed by Rosenberg (1965) and revised into a Chinese version by Li (2002). It has been demonstrated to possess good reliability and validity within the Chinese context in peer-reviewed papers (Yan et al., 2021). The scale consists of 10 items that serve as a self-report measure of an individual’s overall level of self-esteem on a single dimension. Sample items include: “I feel I have many good qualities,” and “I can get things done like most people.” Each item utilizes a 4-point Likert scale, with 1 being “Strongly Disagree” and 4 being “Strongly Agree.” The total score ranges from 4 to 40, with a higher score indicating a higher level of self-esteem. In this study, the Cronbach’s α coefficient for this scale was 0.95.

#### 2.2.4. Prosocial behavior

The measurement for Prosocial Behavior was conducted using the “Prosocial Behavior Scale.” The Prosocial Behavior Scale was developed by Carlo and Randall (2002) and was revised into a Chinese version by Cong (2008). It has been shown to have good reliability and validity within the Chinese context in peer-reviewed papers (Sun et al., 2023). The scale consists of 23 items and serves as a self-report measure of an individual’s prosocial behaviors, encompassing attributes such as openness, anonymity, altruism, obedience, emotionality, and urgency. Sample items include: “I don’t hesitate when others come to me for help,” and “I tend to help people, especially when they’re in a lot of emotional pain.” Each item is rated on a 5-point Likert scale, where 1 signifies “Never” and 5 indicates “Always.” The total score ranges from 23 to 115, with a higher score reflecting a higher level of prosocial behavior. In this study, the Cronbach’s α coefficient for this scale was 0.95.

## 3. Results

### 3.1. Common method bias test

In this research, common method bias was addressed using anonymous surveys and reverse coding of specific items (Zhou and Long, 2004). Concurrently, this research utilized SPSS 25.0 to perform a Harman single-factor analysis for the assessment of common method bias. Results showed that seven factors have eigenvalues exceeding 1, of which the most significant factor accounted for 38.83% of the variance, below the 40% critical threshold. Therefore, there was no common method bias affecting this study.

### 3.2. The correlation between the study variables

This study utilized SPSS 25.0’s Pearson correlation test to compute the mean, standard deviation, and correlations of various variables. As shown in Table 2, the correlation of physical education courses combined with civic education was positively related to cultural confidence (*r* = 0.29, *p* < 0.001), self-esteem (*r* = 0.35, *p* < 0.001), and prosocial behavior (*r* = 0.40, *p* < 0.001). Cultural confidence was positively correlated with self-esteem (*r* = 0.61, *p* < 0.001) and prosocial behavior (*r* = 0.58, *p* < 0.001). Moreover, self-esteem was positively correlated with prosocial behavior (*r* = 0.72, *p* < 0.001).

**TABLE 2:** Descriptive statistics and interrelations among all observed variables.

Variables	*M*	SD	LLCI	ULCL	1	2	3	4
1. PECICE	30.83	7.1	14	44	1			
2. Cultural confidence	30.81	6.03	10	42	0.29***	1		
3. Self-esteem	55.7	12.38	13	70	0.35***	0.61***	1	
4. Prosocial behavior	85.92	16.22	38	115	0.40***	0.58***	0.72***	1

*M*, mean; SD, standard deviation; LLCI, lower limit confidence interval; ULCL, upper limit confidence interval; PECICE, physical education courses integrated with civic education.

***p < 0.001.

### 3.3. The chain-mediating effect analysis

**FIGURE 1:**
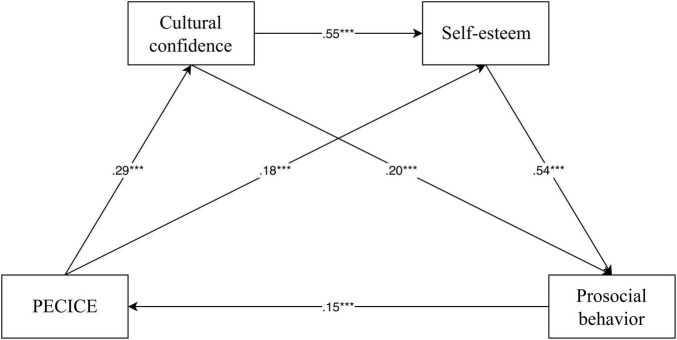
Model of the mediator role of PECICE and prosocial behavior in the relationship between cultural confidence and self-esteem. PECICE, physical education courses integrated with civic education. ***p < 0.001.

Based on the mediation testing method by Wen and Ye (2014), this research used Model 6 from Hayes’s (2013) SPSS plugin PROCESS, with PECICE as the independent variable, prosocial behavior as the dependent variable, and cultural confidence and self-esteem as chained mediating variables. The results of the regression analysis are shown in Table 3. Specifically, Model 1 showed that PECICE positively predicted cultural confidence (β = 0.29, *p* < 0.001), supporting hypothesis 1. Model 2 showed that PECICE positively predicted self-esteem (β = 0.18, *p* < 0.001), and cultural confidence was a significant and positive predictor of self-esteem (β = 0.55, *p* < 0.001). Model 3 showed that PECICE positively predicted prosocial behavior (β = 0.15, *p* < 0.001), cultural confidence was a significant and positive predictor of prosocial behavior (β = 0.20, *p* < 0.001), and self-esteem was a significant and positive predictor of prosocial behavior (β = 0.54, *p* < 0.001). Concurrently, it was found that all the standardized path coefficients in the model were significant (Figure 1).

**TABLE 3:** The chain mediation model from PECICE to prosocial behavior.

	Model 1				Model 2				Model 3			
	Cultural confidence				Self-esteem				Prosocial behavior			
	β	*B*	SE	*t*	β	*B*	SE	*t*	β	*B*	SE	*t*
PECICE	0.29	0.25	0.47	15.31***	0.18	0.32	0.08	3.90***	0.15	0.35	0.09	3.77***
Cultural confidence					0.55	1.13	0.09	11.67***	0.20	0.56	0.13	4.30***
Self-esteem									0.54	0.71	0.06	10.94***
*R* ^2^	0.08				0.40				0.57			
*F*	28.32				98.12				131.55			

PECICE, physical education courses integrated with civic education.

***p < 0.001.

This study further employed the bias-corrected non-parametric percentile Bootstrap method to test the mediation effects, as shown in Table 4. The indirect effect of the pathway with cultural confidence as a mediator was 0.14, accounting for 15.22% of the total effect. The indirect effect of the pathway with self-esteem as a mediator was 0.22, accounting for 23.91% of the total effect. The indirect effect of the pathway with both cultural confidence and self-esteem as mediators was 0.2, accounting for 21.74% of the total effect. In summary, the 95% confidence intervals for all three mediation pathways did not include 0, indicating a significant mediation effect. Hypotheses 2–4 were supported.

**TABLE 4:** Bootstrap mediating effects of PECICE and prosocial behavior.

Paths	Effect	BootSE	BootLLCI	BootULCL	Percentage (%)
Total effect	0.92	0.12	0.68	1.16	
Direct effect	0.34	0.09	0.16	0.53	38.04
Indirect effect	0.57	0.08	0.40	0.75	61.96
Path a: PECICE → CC → PB	0.14	0.04	0.06	0.23	15.22
Path b: PECICE → SE → PB	0.22	0.06	0.11	0.36	23.91
Path c: PECICE → CC → SE → PB	0.2	0.04	0.11	0.30	21.74

PECICE, physical education courses integrated with civic education; CC, cultural confidence; SE, self-esteem; PB, prosocial behavior.

***p < 0.001.

## 4. Discussion

This study explored the influence of PECICE on university students’ prosocial behavior, with a specific focus on the mediating roles of cultural confidence and self-esteem. The data revealed a significant positive correlation between PECICE and the factors of cultural confidence, self-esteem, and prosocial behavior. Moreover, PECICE emerged as a predictor of these three factors. Notably, both cultural confidence and self-esteem served as mediators in the connection between PECICE and prosocial behavior. These findings shed light on the mechanisms explaining “why” and “how” PECICE shapes the prosocial behaviors of university students.

### 4.1. PECICE and prosocial behavior

The research findings aligned with hypothesis 1, suggesting that PECICE significantly predicts the prosocial behavior of university students. These results resonate with prior studies (Liu and Zhou, 2021) that highlight the constructive influence of civic education on prosocial behavior. By extending this understanding, our research demonstrated the affirmative impact of PECICE on university students’ prosocial behavior. To discern the underlying reasons, one must distinguish between the explicit nature of standard civic education and the implicit modality of PECICE (Gao and Zong, 2017). Physical education naturally provides a conducive environment for students to interact, adopt various social roles, and acquire essential social skills like tolerance and respect (Bailey et al., 2009). These interactions can be crucial in nurturing prosocial behavior. Moreover, when civic education gets interwoven with physical activities, students encounter civic education elements in a more dynamic setting, prompting active engagement (Fu and Ding, 2008). Thus, PECICE emerges as an effective tool to foster prosocial behavior in university students. Given this, educators in physical education should emphasize the integration of civic education aspects in their curricula to further facilitate the evolution of prosocial behaviors. By bridging theory with empirical research, this study enriches the discourse on the confluence of physical education and civic education.

### 4.2. The mediating effect of cultural confidence

The research findings support hypothesis 2, asserting that cultural confidence mediates the relationship between PECICE and university students’ prosocial behavior. This aligns with prior research (Zhang et al., 2020), suggesting that acknowledging group achievements spurs prosocial behavior. In essence, as appreciation for group accomplishments deepens, it enhances the group members’ sense of belonging, making them more likely to support their peers (Zhang et al., 2020). Our study further underscores the potential of PECICE to stimulate prosocial behavior by amplifying cultural confidence. Engaging in PECICE likely provides individuals with a more profound appreciation and association with their cultural and social roots (Ming, 2021). Such a deep-seated sense of cultural identity intensifies their affiliation with the group, which in turn, motivates them toward more proactive prosocial actions (Zhang et al., 2020). In summary, PECICE has the potential to accentuate prosocial behavior in university students via the avenue of cultural confidence. Thus, when implementing PECICE, physical education instructors should prioritize nurturing a robust cultural identity to cultivate prosocial behavior among students.

### 4.3. The mediating effect of self-esteem

The research findings support hypothesis 3, suggesting that self-esteem mediates the relationship between PECICE and university students’ prosocial behavior. Existing studies have demonstrated that elevated self-esteem tends to enhance prosocial behavior (Yan, 2021, 2022). Building on this, our research reveals that PECICE can nurture prosocial behavior by bolstering self-esteem. Physical education, inherently emphasizing teamwork, offers avenues for enhancing individual self-esteem (Bailey et al., 2009). Through teamwork, individuals engage collaboratively toward achieving common goals. Experiencing collective achievements, such as successfully completing tasks or winning competitions, instills a sense of pride and fulfillment (Mossholder et al., 1982). Such accomplishments amplify self-esteem by making individuals recognize their valuable contributions to the team’s success (Mossholder et al., 1982). Moreover, individuals with heightened self-esteem often navigate social relationships more positively, stemming from their intrinsic confidence and contentment (Harris and Orth, 2020). This inherent confidence emboldens them to assist, share, and endorse others (Fu et al., 2017). In conclusion, PECICE holds the potential to foster prosocial behavior among university students by enhancing self-esteem. Thus, when applying PECICE, physical education instructors should prioritize nurturing self-esteem through teamwork to catalyze prosocial behavior development.

### 4.4. The chain mediated effect of cultural confidence and self-esteem

The research findings support hypothesis 4, suggesting a chain mediation role of cultural confidence and self-esteem between PECICE and university students’ prosocial behavior. Prior studies indicate a correlation between cultural confidence and self-esteem (Yu et al., 2018). Specifically, individuals with profound awareness and appreciation of their cultural heritage often express heightened self-worth and identity. Such cultural identification boosts their sense of belonging, which consequently augments self-esteem (Yu et al., 2018). Building on this understanding, our study affirms that PECICE influences prosocial behaviors by reinforcing both cultural confidence and self-esteem. PECICE enables students to engage in team dynamics and competition, while simultaneously immersing them in the richness of their cultural lineage (Zhang et al., 2020). This immersive cultural experience solidifies cultural identification, thereby amplifying self-esteem (Yu et al., 2018). Furthermore, the collaborative essence of sports activities in PECICE promotes interpersonal communication and cooperation, fostering positive social relationships and cultivating prosocial behavior (Bailey et al., 2009; Yan, 2021, 2022). Consequently, PECICE offers a holistic learning environment that advances prosocial behavior through the dual pillars of cultural confidence and self-esteem.

In closing, the chain mediation model elucidates the intricate pathways through which PECICE shapes prosocial behavior among university students. These insights underscore the pivotal role of cultural confidence and self-esteem in nurturing positive social behaviors, providing valuable guidance for educational strategies aimed at instilling these qualities in students.

### 4.5. Limitations and future direction

This study acknowledges several limitations that future research can address. Firstly, while questionnaire surveys offer valuable data, they limit our ability to ascertain causal relationships between variables and may sometimes provide incomplete information. Future studies could employ longitudinal or experimental designs to substantiate and expand upon our findings. Secondly, despite our attempts to mitigate common method bias, inherent limitations in self-reported questionnaires, such as memory discrepancies, social desirability, and certain response tendencies, could introduce bias. Thus, future research might benefit from integrating diverse data sources to attain a comprehensive view. Thirdly, our limited sample size, stemming from the early adoption phase of PECICE in select Chinese universities, calls for prudence in generalizing the findings. It would be worthwhile for future endeavors to engage larger and more diverse samples. Fourthly, the study’s focus on Chinese public universities means its findings might not seamlessly apply to Chinese private universities or institutions abroad. Fifthly, the measurement of PECICE probes into participants’ subjective perceptions regarding PECICE rather than scrutinizing the direct effectiveness of the classroom milieu. Therefore, perceptions of PECICE could be influenced by some participant’s characteristics and not only the objective content of PECICE. Future research could explore the objective attributes of PECICE by feedback from multiple sources, thereby attenuating perceptual biases and facilitating a more exhaustive insight into the classroom environment and its ensuing psychosocial implications. Moreover, variations might emerge in PECICE implementations across different Chinese provinces. Upcoming research should delve into the nuances between private Chinese institutions, international counterparts, and inter-provincial differences in China. Lastly, implementing PECICE in institutions championing open discourse may stir ideological debates. Thus, while interpreting the results, a discerning lens is required. In summary, this study paves the way for understanding PECICE’s implications, inspiring more academic institutions to consider its adoption and encouraging continued research in the domain.

## 5. Conclusion

This study indicates that PECICE has a positive impact on the prosocial behavior of college students. Cultural confidence and self-esteem have a chain-mediating effect on the relationship between PECICE and prosocial behavior. These two variables hold significant practical implications for enhancing the prosocial behavior of university students. Thus, this study provides insights for the establishment of PECICE in universities.

## Data availability statement

The raw data supporting the conclusions of this article will be made available by the authors, without undue reservation.

## Ethics statement

The studies involving human participants were reviewed and approved by the Ethics Committee of the Hunan Normal University. The patients/participants provided their written informed consent to participate in this study.

## Author contributions

GL and YTZ conceived the study and drafted the manuscript. NA and ZD collected and analyzed the data. JD and CH conceived the study design and assisted in revising the manuscript. ZZ obtained, analyzed, and parsed data for the modification process of this work. YHZ provided the conceptualization and design in the modification part of this work. All authors contributed to the article and approved the submitted version.
