# Corrigendum: The influence of physical education courses integrated with civic education on prosocial behavior among college students: the chain mediating effect of cultural confidence and self-esteem

**DOI:** 10.3389/fpsyg.2024.1387282

**Published:** 2024-03-12

**Authors:** Changchang Huang, Geng Li, Yuantong Zhang, Nalatporn Aphichaithawon, Zhile Deng, Zhihua Zhang, Yihan Zhang, Jianjun Ding

**Affiliations:** ^1^Hunan Sany Polytechnic College, Changsha, China; ^2^College of Physical Education, Hunan Normal University, Changsha, China; ^3^Rausser College of Natural Resources, University of California, Berkeley, Berkeley, CA, United States; ^4^Shaoyang No. 10 Middle School, Shaoyang, China; ^5^School of Educational Science, Hunan Normal University, Changsha, China; ^6^College of Marxism, Hunan Normal University, Changsha, China

**Keywords:** physical education, prosocial behavior, college student, cultural confidence, self-esteem

In the published article, there was an error in affiliation 1 as published. Instead of “Hunan Trinity Industrial Vocational and Technical College, Changsha, China” it should be “Hunan Sany Polytechnic College, Changsha, China.” Affiliation 1 has been updated.

The authors apologize for this error and state that this does not change the scientific conclusions of the article in any way. The original article has been updated.

